# 2,2-Dimethyl-5-(1-naphthyl­amino­methyl­ene)-1,3-dioxane-4,6-dione

**DOI:** 10.1107/S1600536809033984

**Published:** 2009-08-29

**Authors:** Zhi Li, Rui Li, Zhen-Yu Ding

**Affiliations:** a State Key Laboratory of Biotherapy, West China Hospital, Sichuan University, Chengdu 610041, People’s Republic of China

## Abstract

The benzyl ring of the title compound, C_17_H_15_NO_4_, is twisted away from the plane defined by five atoms of the dioxane ring by 34.83 (4)°. The dioxane ring exhibits a half-boat conformation, with the C atom between the dioxane O atoms 0.571 (8) Å out of the plane through the remainder of the ring. An intra­molecular N—H⋯O hydrogen bond may contribute to the stabilization of the planar conformation of the mol­ecule. In the crystal, inversion dimers linked by pairs of C—H⋯O bonds occur.

## Related literature

For the synthesis of related compounds, see: Cassis *et al.* (1985 ▶). For the pharmacological activity of 4(1*H*)-quinolone structures, see: Ruchelman *et al.* (2003 ▶).
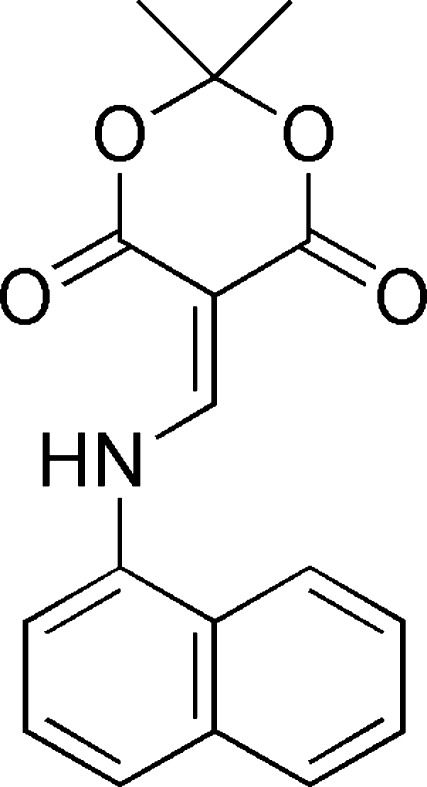


## Experimental

### 

#### Crystal data

C_17_H_15_NO_4_
*M* *_r_* = 297.30
Triclinic, 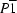

*a* = 7.4696 (11) Å
*b* = 8.0805 (12) Å
*c* = 12.1240 (18) Å
α = 98.601 (2)°
β = 96.428 (2)°
γ = 92.198 (2)°
*V* = 717.87 (18) Å^3^
*Z* = 2
Mo *K*α radiation
μ = 0.10 mm^−1^
*T* = 153 K
0.25 × 0.20 × 0.20 mm

#### Data collection

Bruker SMART CCD area-detector diffractometer
Absorption correction: none
4513 measured reflections
3194 independent reflections
2571 reflections with *I* > 2σ(*I*)
*R*_int_ = 0.014

#### Refinement

*R*[*F* ^2^ > 2σ(*F* ^2^)] = 0.040
*wR*(*F* ^2^) = 0.110
*S* = 1.03
3194 reflections
206 parameters
H atoms treated by a mixture of independent and constrained refinement
Δρ_max_ = 0.18 e Å^−3^
Δρ_min_ = −0.16 e Å^−3^

### 

Data collection: *SMART* (Bruker, 2001 ▶); cell refinement: *SAINT* (Bruker, 2000 ▶); data reduction: *SAINT*; program(s) used to solve structure: *SHELXS97* (Sheldrick, 2008 ▶); program(s) used to refine structure: *SHELXL97* (Sheldrick, 2008 ▶); molecular graphics: *ORTEP-3 for Windows* (Farrugia, 1997 ▶); software used to prepare material for publication: *SHELXL97* and *PLATON* (Spek, 2009 ▶).

## Supplementary Material

Crystal structure: contains datablocks I, global. DOI: 10.1107/S1600536809033984/ez2178sup1.cif

Structure factors: contains datablocks I. DOI: 10.1107/S1600536809033984/ez2178Isup2.hkl

Additional supplementary materials:  crystallographic information; 3D view; checkCIF report

## Figures and Tables

**Table 1:** Hydrogen-bond geometry (Å, °)

*D*—H⋯*A*	*D*—H	H⋯*A*	*D*⋯*A*	*D*—H⋯*A*
N1—H1⋯O3	0.920 (18)	1.982 (18)	2.7130 (16)	135.2 (15)
C1—H1*C*⋯O4^i^	0.98	2.60	3.3709 (19)	136

Symmetry code: (i) 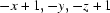.

**Fig. 1.**
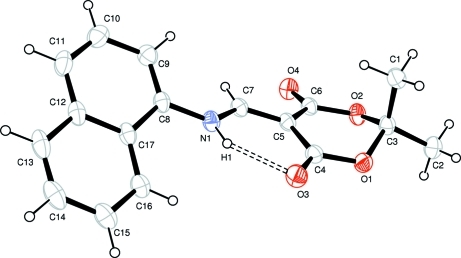
The molecular structure of the title compound, with displacement ellipsoids drawn at the 30% probability level. The hydrogen bond is shown by a dashed line.

## supplementary crystallographic information

### Comment

4(1*H*)-Quinolone structures have long attracted pharmacological interest as anticancer agents, anti-malarial agents and reversible (H+/K+) ATPase inhibitors (Ruchelman *et al.*, 2003). Thermolysis of 5-arylaminomethylene-2,2-dimethyl-1,3-dioxane-4,6-diones is an effective method to synthesize 4(1*H*)quinolone derivatives (Cassis *et al.*, 1985).

The benzyl ring is twisted away from the plane defined by the dioxane ring by 34.83 (4)°. In turn, the dioxane ring of the title compound exhibits an envelope conformation, in which the flap atom, the C atom between the dioxane oxygen atoms, is -0.571 (8) Å out of the plane. An intramolecular N—H···O hydrogen bond (Table 1) could lead to the dioxane ring and the aminomethylene group taking up their planar conformation.

### Experimental

An ethanol solution (50 ml) of 2,2-dimethyl-1,3-dioxane-4,6-dione (Meldrum's acid) (1.44 g, 0.01 mol) and methylorthoformate (1.27 g, 0.012 mol) was heated to reflux for 2 h, then the naphthalen-1-amine (1.43 g, 0.01 mol) was added into the above solution. The mixture was heated under reflux for another 8 h and then filtered. Single crystals were obtained from the filtrate after 2 days.

### Refinement

The imino H atom was located in a difference Fourier map and refined isotropically. Other H atoms were positioned geometrically with C—H = 0.93 (aromatic) or 0.96 Å (methyl), and refined using a riding model with *U*_ĩso_(H) = 1.5*U*_eq_(C) for methyl and 1.2*U*_eq_(C) for the others.

### Crystal data

**Table:**

C_17_H_15_NO_4_	*Z* = 2
*M**_r_* = 297.30	*F*(000) = 312
Triclinic, *P*1	*D*_x_ = 1.375 Mg m^−^^3^
*a* = 7.4696 (11) Å	Mo *K*α radiation, λ = 0.71073 Å
*b* = 8.0805 (12) Å	Cell parameters from 2197 reflections
*c* = 12.1240 (18) Å	θ = 2.6–27.5°
α = 98.601 (2)°	µ = 0.10 mm^−^^1^
β = 96.428 (2)°	*T* = 153 K
γ = 92.198 (2)°	Block, colourless
*V* = 717.87 (18) Å^3^	0.25 × 0.20 × 0.20 mm

### Data collection

**Table:**

Bruker SMART CCD area-detector diffractometer	2571 reflections with *I* > 2σ(*I*)
Radiation source: fine-focus sealed tube	*R*_int_ = 0.014
graphite	θ_max_ = 27.6°, θ_min_ = 2.6°
φ and ω scans	*h* = −9→6
4513 measured reflections	*k* = −10→10
3194 independent reflections	*l* = −15→15

### Refinement

**Table:**

Refinement on *F*^2^	Secondary atom site location: difference Fourier map
Least-squares matrix: full	Hydrogen site location: mixed
*R*[*F*^2^ > 2σ(*F*^2^)] = 0.040	H atoms treated by a mixture of independent and constrained refinement
*wR*(*F*^2^) = 0.110	*w* = 1/[σ^2^(*F*_o_^2^) + (0.0462*P*)^2^ + 0.1278*P*] where *P* = (*F*_o_^2^ + 2*F*_c_^2^)/3
*S* = 1.03	(Δ/σ)_max_ < 0.001
3194 reflections	Δρ_max_ = 0.18 e Å^−^^3^
206 parameters	Δρ_min_ = −0.16 e Å^−^^3^
0 restraints	Extinction correction: *SHELXL97* (Sheldrick, 2008), Fc^*^=kFc[1+0.001xFc^2^λ^3^/sin(2θ)]^-1/4^
Primary atom site location: structure-invariant direct methods	Extinction coefficient: 0.204 (9)

### Special details

**Table:**

Geometry. All e.s.d.'s (except the e.s.d. in the dihedral angle between two l.s. planes) are estimated using the full covariance matrix. The cell e.s.d.'s are taken into account individually in the estimation of e.s.d.'s in distances, angles and torsion angles; correlations between e.s.d.'s in cell parameters are only used when they are defined by crystal symmetry. An approximate (isotropic) treatment of cell e.s.d.'s is used for estimating e.s.d.'s involving l.s. planes.
Refinement. Refinement of *F*^2^ against ALL reflections. The weighted *R*-factor *wR* and goodness of fit *S* are based on *F*^2^, conventional *R*-factors *R* are based on *F*, with *F* set to zero for negative *F*^2^. The threshold expression of *F*^2^ > σ(*F*^2^) is used only for calculating *R*-factors(gt) *etc*. and is not relevant to the choice of reflections for refinement. *R*-factors based on *F*^2^ are statistically about twice as large as those based on *F*, and *R*- factors based on ALL data will be even larger.

### Fractional atomic coordinates and isotropic or equivalent isotropic displacement parameters (Å^2^)

**Table:**

	*x*	*y*	*z*	*U*_iso_*/*U*_eq_	
O1	0.75507 (13)	0.44446 (12)	0.48110 (8)	0.0482 (3)	
O2	0.61634 (12)	0.29559 (12)	0.60505 (7)	0.0468 (3)	
O3	0.66547 (15)	0.40648 (13)	0.29855 (8)	0.0567 (3)	
O4	0.36615 (13)	0.13711 (12)	0.54766 (8)	0.0501 (3)	
N1	0.41749 (15)	0.15238 (15)	0.21325 (9)	0.0431 (3)	
H1	0.496 (3)	0.236 (2)	0.2012 (15)	0.074 (5)*	
C1	0.9036 (2)	0.21096 (19)	0.54644 (13)	0.0529 (4)	
H1A	0.9247	0.1511	0.6109	0.079*	
H1B	1.0193	0.2541	0.5281	0.079*	
H1C	0.8432	0.1340	0.4816	0.079*	
C2	0.8669 (2)	0.4816 (2)	0.67397 (13)	0.0583 (4)	
H2A	0.7830	0.5704	0.6887	0.087*	
H2B	0.9809	0.5306	0.6571	0.087*	
H2C	0.8895	0.4262	0.7404	0.087*	
C3	0.78588 (18)	0.35520 (17)	0.57518 (11)	0.0433 (3)	
C4	0.64805 (18)	0.36396 (16)	0.38885 (11)	0.0420 (3)	
C5	0.51881 (17)	0.23873 (16)	0.40821 (10)	0.0384 (3)	
C6	0.49111 (17)	0.21600 (16)	0.52166 (10)	0.0393 (3)	
C7	0.41364 (17)	0.14044 (16)	0.32061 (10)	0.0401 (3)	
H7	0.3322	0.0579	0.3385	0.048*	
C8	0.32676 (17)	0.04147 (17)	0.12053 (10)	0.0412 (3)	
C9	0.2809 (2)	−0.12053 (18)	0.12899 (12)	0.0502 (3)	
H9	0.3068	−0.1605	0.1986	0.060*	
C10	0.1953 (2)	−0.2285 (2)	0.03460 (14)	0.0598 (4)	
H10	0.1584	−0.3399	0.0416	0.072*	
C11	0.1648 (2)	−0.1750 (2)	−0.06667 (13)	0.0593 (4)	
H11	0.1097	−0.2505	−0.1301	0.071*	
C12	0.21389 (18)	−0.0086 (2)	−0.07868 (11)	0.0495 (4)	
C13	0.1908 (2)	0.0502 (3)	−0.18422 (12)	0.0624 (5)	
H13	0.1415	−0.0247	−0.2495	0.075*	
C14	0.2376 (2)	0.2106 (3)	−0.19338 (13)	0.0663 (5)	
H14	0.2247	0.2460	−0.2650	0.080*	
C15	0.3050 (2)	0.3242 (2)	−0.09805 (13)	0.0608 (4)	
H15	0.3325	0.4378	−0.1047	0.073*	
C16	0.3315 (2)	0.2734 (2)	0.00486 (12)	0.0513 (4)	
H16	0.3775	0.3521	0.0690	0.062*	
C17	0.29151 (17)	0.10513 (18)	0.01706 (10)	0.0428 (3)	

### Atomic displacement parameters (Å^2^)

**Table:**

	*U*^11^	*U*^22^	*U*^33^	*U*^12^	*U*^13^	*U*^23^
O1	0.0558 (6)	0.0465 (5)	0.0405 (5)	−0.0043 (4)	−0.0028 (4)	0.0094 (4)
O2	0.0450 (5)	0.0633 (6)	0.0309 (5)	−0.0001 (4)	0.0023 (4)	0.0057 (4)
O3	0.0686 (7)	0.0633 (6)	0.0400 (6)	−0.0081 (5)	0.0034 (5)	0.0195 (5)
O4	0.0499 (6)	0.0620 (6)	0.0394 (5)	−0.0027 (5)	0.0111 (4)	0.0081 (4)
N1	0.0441 (6)	0.0532 (7)	0.0315 (6)	0.0003 (5)	0.0015 (4)	0.0076 (5)
C1	0.0452 (8)	0.0568 (8)	0.0550 (9)	0.0065 (6)	−0.0014 (6)	0.0080 (7)
C2	0.0610 (9)	0.0622 (9)	0.0457 (8)	−0.0021 (7)	−0.0061 (7)	−0.0001 (7)
C3	0.0440 (7)	0.0503 (7)	0.0346 (7)	0.0002 (6)	0.0001 (5)	0.0074 (5)
C4	0.0478 (7)	0.0433 (7)	0.0349 (7)	0.0044 (5)	0.0014 (5)	0.0081 (5)
C5	0.0398 (7)	0.0448 (7)	0.0311 (6)	0.0056 (5)	0.0023 (5)	0.0079 (5)
C6	0.0398 (7)	0.0447 (7)	0.0335 (6)	0.0069 (5)	0.0037 (5)	0.0055 (5)
C7	0.0391 (7)	0.0483 (7)	0.0341 (6)	0.0060 (5)	0.0047 (5)	0.0087 (5)
C8	0.0357 (6)	0.0547 (8)	0.0322 (6)	0.0057 (5)	0.0033 (5)	0.0030 (5)
C9	0.0539 (8)	0.0557 (8)	0.0414 (7)	0.0080 (6)	0.0071 (6)	0.0062 (6)
C10	0.0617 (10)	0.0548 (9)	0.0594 (10)	0.0020 (7)	0.0080 (7)	−0.0026 (7)
C11	0.0517 (9)	0.0699 (10)	0.0475 (9)	0.0058 (7)	−0.0028 (7)	−0.0136 (7)
C12	0.0386 (7)	0.0714 (9)	0.0354 (7)	0.0127 (6)	0.0010 (5)	−0.0017 (6)
C13	0.0523 (9)	0.0978 (13)	0.0327 (7)	0.0195 (8)	−0.0036 (6)	−0.0017 (8)
C14	0.0590 (10)	0.1060 (14)	0.0386 (8)	0.0215 (9)	0.0055 (7)	0.0229 (9)
C15	0.0567 (9)	0.0829 (11)	0.0477 (9)	0.0095 (8)	0.0052 (7)	0.0246 (8)
C16	0.0489 (8)	0.0663 (9)	0.0391 (7)	0.0034 (7)	0.0023 (6)	0.0119 (6)
C17	0.0343 (6)	0.0622 (8)	0.0315 (6)	0.0089 (6)	0.0040 (5)	0.0044 (6)

### Geometric parameters (Å, °)

**Table:**

O1—C4	1.3622 (15)	C7—H7	0.9500
O1—C3	1.4403 (15)	C8—C9	1.362 (2)
O2—C6	1.3632 (15)	C8—C17	1.4271 (18)
O2—C3	1.4405 (16)	C9—C10	1.405 (2)
O3—C4	1.2142 (15)	C9—H9	0.9500
O4—C6	1.2072 (15)	C10—C11	1.360 (2)
N1—C7	1.3229 (16)	C10—H10	0.9500
N1—C8	1.4170 (16)	C11—C12	1.413 (2)
N1—H1	0.920 (18)	C11—H11	0.9500
C1—C3	1.510 (2)	C12—C17	1.4204 (19)
C1—H1A	0.9800	C12—C13	1.425 (2)
C1—H1B	0.9800	C13—C14	1.353 (3)
C1—H1C	0.9800	C13—H13	0.9500
C2—C3	1.5063 (19)	C14—C15	1.396 (2)
C2—H2A	0.9800	C14—H14	0.9500
C2—H2B	0.9800	C15—C16	1.367 (2)
C2—H2C	0.9800	C15—H15	0.9500
C4—C5	1.4345 (18)	C16—C17	1.414 (2)
C5—C7	1.3743 (17)	C16—H16	0.9500
C5—C6	1.4508 (17)		
			
C4—O1—C3	116.84 (10)	N1—C7—H7	117.6
C6—O2—C3	118.48 (10)	C5—C7—H7	117.6
C7—N1—C8	126.21 (12)	C9—C8—N1	121.29 (12)
C7—N1—H1	113.8 (11)	C9—C8—C17	121.54 (12)
C8—N1—H1	119.7 (11)	N1—C8—C17	117.13 (12)
C3—C1—H1A	109.5	C8—C9—C10	119.85 (14)
C3—C1—H1B	109.5	C8—C9—H9	120.1
H1A—C1—H1B	109.5	C10—C9—H9	120.1
C3—C1—H1C	109.5	C11—C10—C9	120.64 (15)
H1A—C1—H1C	109.5	C11—C10—H10	119.7
H1B—C1—H1C	109.5	C9—C10—H10	119.7
C3—C2—H2A	109.5	C10—C11—C12	120.82 (14)
C3—C2—H2B	109.5	C10—C11—H11	119.6
H2A—C2—H2B	109.5	C12—C11—H11	119.6
C3—C2—H2C	109.5	C11—C12—C17	119.39 (13)
H2A—C2—H2C	109.5	C11—C12—C13	122.49 (14)
H2B—C2—H2C	109.5	C17—C12—C13	118.11 (15)
O1—C3—O2	110.06 (10)	C14—C13—C12	121.36 (15)
O1—C3—C2	106.69 (11)	C14—C13—H13	119.3
O2—C3—C2	105.71 (11)	C12—C13—H13	119.3
O1—C3—C1	109.85 (11)	C13—C14—C15	120.28 (15)
O2—C3—C1	110.62 (11)	C13—C14—H14	119.9
C2—C3—C1	113.75 (12)	C15—C14—H14	119.9
O3—C4—O1	118.14 (12)	C16—C15—C14	120.52 (16)
O3—C4—C5	125.52 (12)	C16—C15—H15	119.7
O1—C4—C5	116.31 (11)	C14—C15—H15	119.7
C7—C5—C4	121.36 (11)	C15—C16—C17	120.84 (15)
C7—C5—C6	117.92 (11)	C15—C16—H16	119.6
C4—C5—C6	120.69 (11)	C17—C16—H16	119.6
O4—C6—O2	118.28 (11)	C16—C17—C12	118.73 (13)
O4—C6—C5	125.74 (12)	C16—C17—C8	123.65 (12)
O2—C6—C5	115.95 (11)	C12—C17—C8	117.61 (13)
N1—C7—C5	124.76 (12)		

### Hydrogen-bond geometry (Å, °)

**Table:**

*D*—H···*A*	*D*—H	H···*A*	*D*···*A*	*D*—H···*A*
N1—H1···O3	0.920 (18)	1.982 (18)	2.7130 (16)	135.2 (15)
C1—H1C···O4^i^	0.98	2.60	3.3709 (19)	136

Symmetry codes: (i) −*x*+1, −*y*, −*z*+1.
